# The Aleutians and Beyond: Distribution, Size Composition, and Catch Dynamics of the Aleutian Skate *Bathyraja aleutica* across the North Pacific

**DOI:** 10.3390/ani12243507

**Published:** 2022-12-12

**Authors:** Igor V. Grigorov, Kirill K. Kivva, Alexei M. Orlov

**Affiliations:** 1 Fish Breeding Division, Central Branch of the Federal State-Funded Institution “Glav Basin Department of Fisheries and Conservation of Water Biological Resources”, 117105 Moscow, Russia; 2 Department of Climate and Aquatic Ecosystems Dynamics, Russian Federal Research Institute of Fisheries and Oceanography, 105187 Moscow, Russia; 3 Laboratory of Oceanic Ichthyofauna, Shirshov Institute of Oceanology, Russian Academy of Sciences, 117997 Moscow, Russia; 4 Laboratory of Behavior of Lower Vertebrates, A.N. Severtsov Institute of Ecology and Evolution, Russian Academy of Sciences, 119071 Moscow, Russia; 5 Department of Ichthyology and Hydrobiology, Tomsk State University, 634050 Tomsk, Russia; 6 Department of Ichthyology, Dagestan State University, 367000 Makhachkala, Russia; 7 Laboratory of Marine Biology, Dagestan Federal Research Center, Caspian Institute of Biological Resources, Russian Academy of Sciences, 367000 Makhachkala, Russia

**Keywords:** spatial distribution, vertical distribution, length, body weight, sex ratio, condition factor, Bering Sea, Sea of Okhotsk, Kuril Islands, Aleutian Islands, Gulf of Alaska, British Columbia

## Abstract

**Simple Summary:**

Deep-water skates play an important role as top predators in the North Pacific, yet they are also considered promising targets of bottom-trawl and longline fisheries. Moreover, skates are highly vulnerable to over-fishing due to their very long lifespans, late maturation, small litter size, long incubation period, etc. Despite their ecological and commercial importance, the distribution, basic biological traits, and dynamics of abundance of many deep-water skate species remain poorly understood, information that is critical for their conservation and management. The Aleutian skate *Bathyraja aleutica* is one of the more common deep-water species and is widely distributed across the North Pacific, but it remains largely understudied. In this paper, we compiled and analyzed long-term data records of the Aleutian skate in the North Pacific from various databases, which revealed new information on its spatial and vertical distribution, size composition, reproductive biology, and interannual catch dynamics.

**Abstract:**

The results of long-term (1948–2021) studies on the spatial and vertical distribution, dynamics of abundance, and size composition of the Aleutian skate *Bathyraja aleutica* in the North Pacific Ocean are presented. Maximum densities of this species were characteristic of the eastern Bering Sea slope, off the central Aleutian Islands, consisting of the Pacific waters off southeastern Kamchatka and the northern Kurils, and northeastern Sakhalin. This species was most abundant at depths of 100–600 m; in the cold months, *B. aleutica* migrates to greater depths for over-wintering, and in warm months it feeds at shallower depths. *Bathyraja aleutica* was most common at the bottom, at temperatures around 3 °C. The total length of individuals ranged from 9.6–170 cm, with a predominance of skates with a length of 50–100 cm. Males did not differ significantly from females in body weight and length. The maximum values of the condition factor were typical for the autumn–winter period. Across years, there was an increase in Aleutian skate catch rates from the western Bering Sea and the Sea of Okhotsk, and a decrease in the Pacific waters off the Kuril Islands and Kamchatka, as well as in Alaskan waters.

## 1. Introduction

Skates and rays (Rajiformes) are an important component of the bottom-fish communities of the world’s oceans. In the North Pacific, they are consumers of such ecologically and commercially important species as Pacific herring *Clupea pallasii*, walleye pollock *Gadus chalcogrammus*, flathead flounder *Hippoglossoides elassodon*, yellowfin flounder *Limanda aspera*, rock sole *Lepidopsetta bilineata*, Atka mackerel *Pleurogrammus monopterygius*, Pacific cod *Gadus macrocephalus*, shortraker rockfish *Sebastes borealis*, popeye grenadier *Coryphaenoides cinereus*, commander squid *Berryteuthis magister*, octopus (Octopoda), golden king crab *Lithodes aequispinis*, Tanner crabs *Chionoecetes* spp., and shrimps [1,2,3,4,5,6,7,8].

Skates are important targets of coastal fishing in many countries, especially in Southeast Asia, where their wings are dried for food, and specifically their meat is used for the production of crab sticks [9]. Fishing for skates in the North Pacific is still poorly developed, but quite promising [10,11]. Their biomass within Russian waters only amounts to about 677 thousand tons [12], but in some areas skates make up as much as 10% of the total groundfish biomass, and their abundance has increased markedly in recent years [13]. Technological studies have shown that the meat of skates contains almost a complete set of essential amino acids and is highly suitable for food production [14]. Moreover, the liver of skates is rich in vitamin A, and can serve as raw materials for the production of veterinary and medical oils [15]. Skates have a fairly high cost in Japan, where they are sold frozen at a price of JPY 650 per kg, and chilled at a price of JPY 800–1800 per kg. However, in the markets of the west coast of the USA, frozen skates only cost from USD 0.1 to 0.3 per pound [16]. Fishing for skates and the export of raw materials or other fishery products from Russia to Europe, Asia and America, where they are in high demand, was recognized in the early 1980s [17]. In recent years, several Russian companies that have focused on the supply of fish products to Japanese, Korean, and Chinese markets have also shown an interest in the skate fishery. It is therefore likely that in the following years, the volume of their catch of skates may increase significantly.

Until recently, skate fishing in the North Pacific was unregulated, with no established total allowable catches (TAC) and no catch statistics, since the skates were not retained and were discarded. The situation has changed in recent decades, when TAC of skates differentiated by area and species was introduced for both Russian and American waters [18,19].

The Aleutian skate *Bathyraja aleutica* (Gilbert, 1896) is widespread across the North Pacific Ocean, and it plays an important role in ecosystems [20]. It is a boreal species inhabiting the Okhotsk and Bering Seas, waters off the eastern Kamchatka, Japan, Kuril, and Aleutian Islands, as well as the Gulf of Alaska and along the American and Canadian Pacific coast down to California [12,20,21,22,23,24,25,26,27,28,29,30,31]. The information available from the literature on the distribution and biology of this species is not sufficient to understand its life history cycle, and it has so far been scattered and fragmentary [4,5,6,8,18,20,32,33,34,35,36,37,38,39,40,41,42,43,44,45,46,47,48,49,50,51,52,53,54,55,56,57,58,59,60,61,62,63].

The purpose of this paper is to summarize and analyze long-term data on the spatial and vertical distributions, size composition, biology, and dynamics of abundance of the Aleutian skate in the North Pacific.

## 2. Materials and Methods

**Figure 1:**
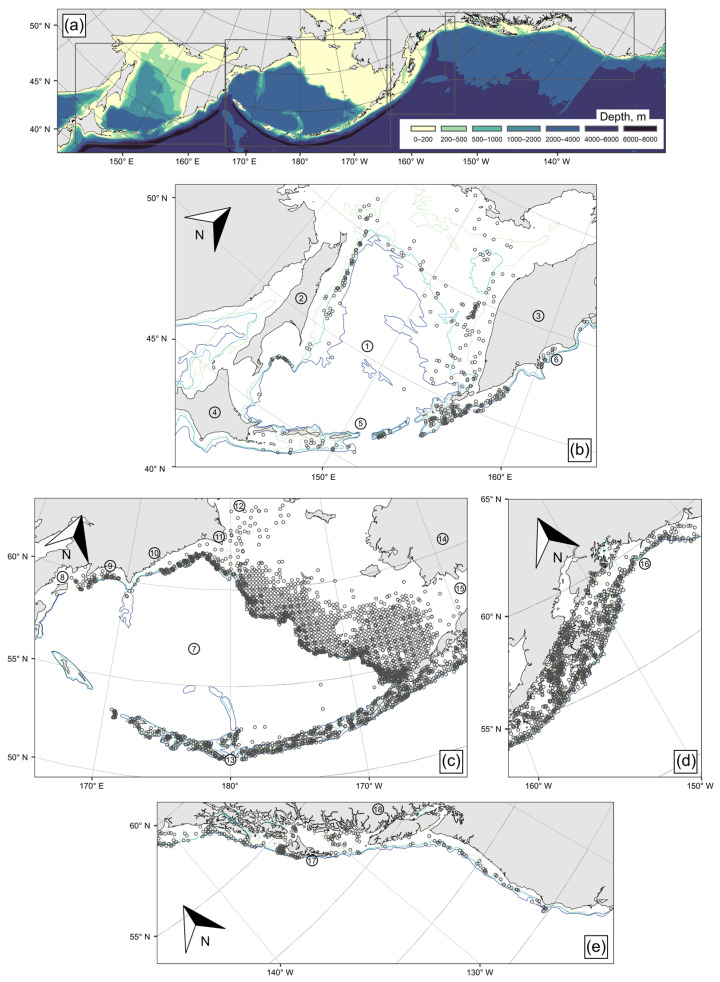
Study area (**a**) and location of trawl stations (small circles) in the survey areas: Sea of Okhotsk and North Pacific waters off Hokkaido, Kuril Islands, and eastern Kamchatka (**b**), Bering Sea (**c**), Gulf of Alaska (**d**), and US and Canada West Coast (**e**). Grey rectangles in (**a**) correspond to panels (**b**–**e**). The numbers in circles indicate the geographical names mentioned in the text: ➀—Sea of Okhotsk, ➁—Sakhalin Island, ➂—Kamchatka Peninsula, ➃—Hokkaido Island, ➄—Kuril Islands, ➅—Kronotsk Bay, ➆—Bering Sea, ➇—Karagin Bay, ➈—Olyutor Bay, ➉—Koryak Coast, ⑪—Navarin Cape, ⑫—Gulf of Anadyr, ⑬—Aleutian Islands, ⑭—Alaska, ⑮—Bristol Bay, ⑯—Gulf of Alaska, ⑰—Haida Gwaii, ⑱—British Columbia.

Data for analysis of *Bathyraja aleutica* population and biological characteristics and trends were obtained from trawl, longline, and trap catches in the North Pacific from 1976 to 2021, obtained mostly by scientific surveys of the Pacific Branch of the Russian Federal Research Institute of Fisheries and Oceanography (TINRO, Vladivostok, Russia), the Alaska Fisheries Science Center (AFSC, Seattle, WA, USA), and the Northwestern Fisheries Science Center (NWFSC, Seattle, WA, USA), as well as by scientific observers on American commercial fishing vessels. In addition, we used the data on Aleutian skate records from ichthyological collections from a number of museums (Table 1). The materials used in this study consist entirely of information and data on those catches in which the Aleutian skate was recorded. Data from the USA bottom-trawl surveys were limited to the spring–summer sampling months of May–October. From the USA commercial fisheries observer data, other than year there was no information on the specific month or season collected, which precluded the data’s full use in analyzing seasonal patterns in Aleutian skate abundance and distribution. In total, the data analyzed consisted of 15,377 catches of the Aleutian skate with the following fishing gear: mid-water, bottom, and pelagic trawls, as well as longlines and traps, including 6746 with records of fishing depth (Figure 1).

To characterize the size composition, measurements of total length (TL) of 3541 individuals were used, including 770 females and 601 males, of which 455 and 376 specimens were weighed, respectively. Analysis of the size and weight composition is presented for the Bering Sea (422 ind.), the Pacific waters off the Kuril Islands and eastern Kamchatka (hereinafter NWPO) (228 ind.), the Sea of Okhotsk (111 ind.), and the Pacific coast of Canada (70 ind.). The analysis of the sex ratio within different size classes was also based on these data. The relationship between total length (TL, cm) and body weight (W, kg) for individuals of both sexes (831 ind.) was estimated from the data on the number of skates caught and their weight in the catch in the case of catching single individuals. The same data are the basis for analysis of Fulton’s condition factor. Maps of spatial distributions were constructed using the GIS and R software.

Visualization of Aleutian skate spatial distribution was performed in R (https://www.R-project.org/, accessed on 24 October 2022). A discrete global hexagonal grid was created using the dggridR package (https://github.com/r-barnes/dggridR/, accessed on 24 October 2022). The distance between the centers of the hexagons was set to about 100 km. The main advantage of this approach is to maintain spatial bins of equal area across a wide range of latitudes and longitudes. Data were averaged over the cells containing >2 data points. For illustrative purposes, data points outside such cells are also shown on the maps. All maps were presented in the North Pole Lambert azimuthal equal-area projection for the Bering Sea (EPSG:3571).

Statistical analysis of the data was performed in R. Welch’s *t*-test was used to compare two independent samples in cases when the distributional assumptions held (e.g., in the case of length-related samples) and when the sample sizes were greater than 40. Prior to performing *t*-tests, outliers were removed according to the interquartile range (IQR) method (threshold values Q_1_ − 1.5 × IQR and Q_3_ + 1.5 × IQR, where Q_1_ and Q_3_ are the first and third quartiles, respectively). In cases when violation of the distributional assumptions was suspected or when the sample size of at least one sample was less than 40, a Wilcoxon rank-sum test (also known as U-test or Mann–Whitney test) was performed. Holm adjustment of *p*-value was used in cases when pairwise comparison was required for both Welch’s *t*-test and Wilcoxon rank-sum test. For the sex ratios, proportion tests (z-tests) were carried out (comparison to 1:1). In order to explore length–weight relationships as well as to compare them for males and females and across different regions, linear models were fitted to the data with and without interaction terms. An alpha level (α) of 0.05 was used in all statistical tests.

## 3. Results

### 3.1. Spatial Distribution of Catches and Their Seasonal and Long-Term Changes

**Figure 2:**
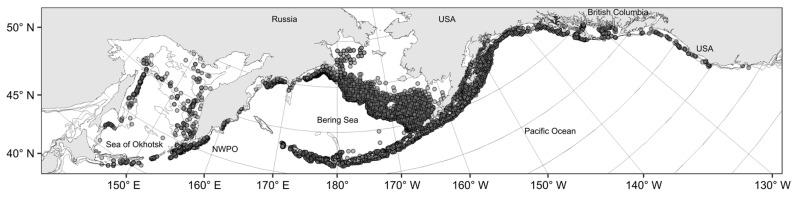
Sites of Aleutian skate *Bathyraja aleutica* captures (●) in the North Pacific Ocean.

The Aleutian skate is widely distributed in the North Pacific, with a known range extension from the northern part of the Gulf of Anadyr in the Bering Sea, to the Pacific coasts of Hokkaido and central California in the south, including the waters of the Bering and Okhotsk Seas (Figure 2). In its distribution, it tends to reside in the lower part of the shelf and the continental slope, and is most often found in the waters off western Kamchatka in the Sea of Okhotsk, the Pacific waters off the northern Kurils, the eastern Bering Sea slope, the Aleutian Islands, and the western part of the Gulf of Alaska.

**Figure 3:**
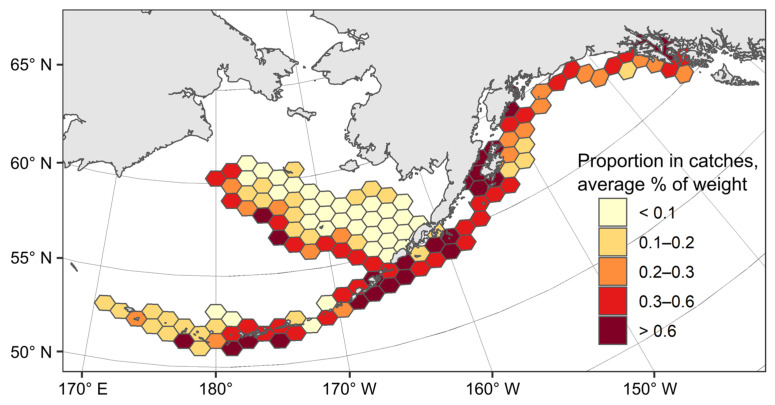
Distribution of catches (proportion in catches, % of weight) of the Aleutian skate *Bathyraja aleutica* in Alaskan waters in 1995–2021 according to data from American observers on board commercial fishing vessels.

According to the USA commercial fishing observer catch data from 1995 to 2021 (Figure 3), the Aleutian skate was the most numerous along the eastern Bering Sea slope, the Aleutian Islands, and the western part of the Gulf of Alaska, where its by-catch exceeded 0.6% of the total catch weight. From other areas in Alaskan waters, the by-catch of the Aleutian skate was considerably lower.

**Figure 4:**
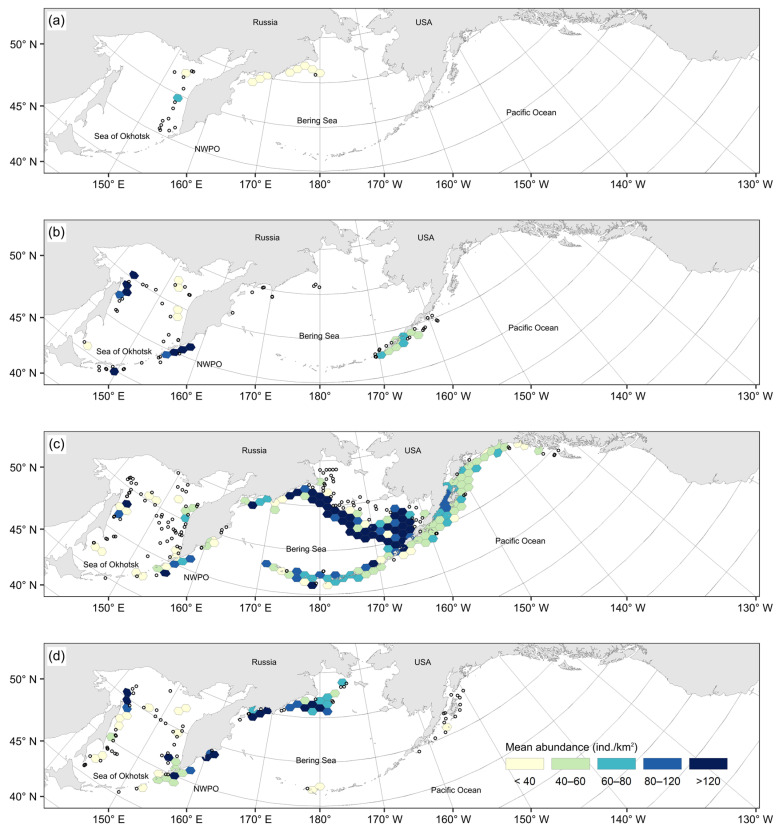
Mean abundance (ind./km^2^) of the Aleutian skate *Bathyraja aleutica* in the North Pacific Ocean in (**a**) December–February, (**b**) March–May, (**c**) June–August, and (**d**) September–November. Small circles mark station locations outside of the hexagonal grid cells with at least three stations (see Methods).

Within-year seasonal variability in the spatial distribution of the Aleutian skate catch varied significantly. In winter months (December–February), as a whole, their values were minimal across the entire survey area (Figure 4a). Maximum catches during this period were recorded from only a few local areas, i.e., in the Pacific waters off the northern Kurils, off the central Aleutian Islands, the central part of the Koryak coast in the western Bering Sea, and in the southern part of the Bristol Bay of the eastern Bering Sea. During the spring, catches of Aleutian skates increased significantly across almost the entire surveyed area (Figure 4b). The highest densities were observed near northeastern Sakhalin, southwestern Kamchatka, the northern part of Karagin Bay, Olyutor Bay, the eastern part of the Koryak coast, along the entire eastern Bering Sea slope, along almost the entire Aleutian Islands, and in the western part of the Gulf of Alaska. In summer, the spatial distribution pattern of Aleutian skate catches changed little (Figure 4c), with maximum catches observed off northeastern Sakhalin, in the western part of the Gulf of Alaska, along the eastern Bering Sea slope, and in the northern part of Karagin Bay. At the same time, the density of aggregations along the Aleutian Islands decreased, and dense schoolings remained off the most western part. Maximum catches from the waters off western Kamchatka shifted to the Pacific waters off the northern Kurils and southwestern Kamchatka. Also, the maximum catches were recorded locally to the east of the southern Kurils. In the autumn, the density of aggregations and the areas with maximum catches significantly decreased (Figure 4d). Dense accumulations were observed in the waters off northeastern Sakhalin, in Olyutor Bay, in the northern part of Karagin Bay and along the eastern Bering Sea slope. In the waters off the northern Kuril and Aleutian Islands, maximum catches were occasional and recorded locally.

**Figure 5:**
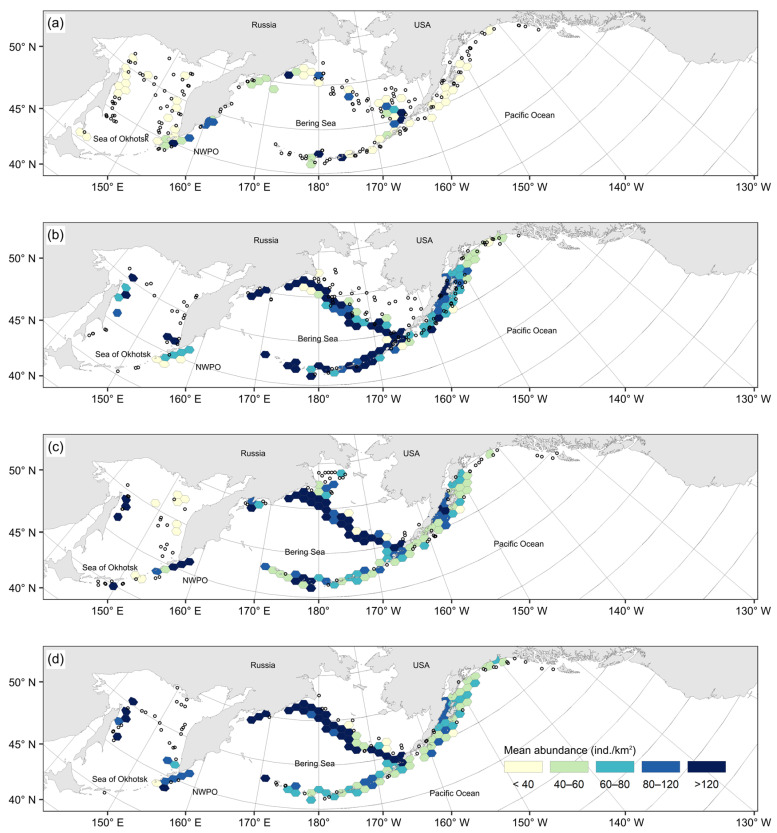
Mean abundance (ind./km^2^) of the Aleutian skate *Bathyraja aleutica* in the North Pacific Ocean in 1976–1990 (**a**), 1991–2000 (**b**), 2001–2010 (**c**), and 2011–2019 (**d**). Small circles mark station locations outside of the hexagonal grid cells with at least three stations (see Methods).

Long-term interannual variation in the distribution of Aleutian skate catches was observed. From the 1990s, data on bottom-trawl surveys were available only from the Russian Exclusive Economic Zone (EEZ) (Figure 5a). During this period, catches of the species considered were recorded only in the western Bering Sea and in the Sea of Okhotsk along the coast of western Kamchatka and the northern Kurils. The maximum mean density of schoolings did not exceed 60–80 ind./km^2^ and was recorded only in a local area of the central part of western Kamchatka. In 1991–2000s, catches of the Aleutian skate were recorded across the entire research area (Figure 5b). In Russian waters, it was observed from the southern Kurils in the south to Navarin Cape in the north. In the Sea of Okhotsk, catches were recorded along the entire eastern coast of Sakhalin, in the northern part of the sea and along western Kamchatka. In American waters, catches were recorded from the central Aleutian Islands to the western part of the Gulf of Alaska. Maximum mean catches exceeding 120 ind./km^2^ were recorded off northeastern Sakhalin, the southern and northern Kurils, and southeastern Kamchatka. In 2001–2010, catches of the Aleutian skate in the study area were distributed most widely (Figure 5c). In Russian waters, it was found from the southern Kuril Islands in the south to the northern part of the Gulf of Anadyr in the north. In the Sea of Okhotsk, it was observed from nearly every site, with the exception of deep-water basins in the central and southwestern parts of the sea. In North American waters, it was registered in catches along the entire Aleutian Islands, in the Gulf of Alaska, and in waters off British Columbia south to Haida Gwaii. However, the Aleutian skate was most numerous along the eastern Bering Sea slope, where the maximum mean catches exceeded 120 ind./km^2^. Single highest-density catches were also recorded off the eastern and central Aleutian Islands, in the northern part of Karagin Bay, in the Pacific waters off the northern Kurils, and off northeastern Sakhalin. After 2011, the overall distribution of the Aleutian skate in the North Pacific significantly decreased, especially in the waters off Alaska (Figure 5d). In Russian waters, the pattern of catch distribution practically did not change. Schoolings with maximum densities were still observed near northeastern Sakhalin and the northern Kurils, in the northern part of Karagin Bay, and along the Koryak coast. In addition, local areas with the maximum density aggregations were located in the waters off southwestern Kamchatka in the Kronotsk and Olyutor bays. In comparison with the previous period, there were practically no catches of this species along the Aleutian Islands, in most of the eastern Bering Sea slope waters, in the eastern part of the Gulf of Alaska, and in the waters off British Columbia.

### 3.2. Distribution Depending on Bottom Temperatures

**Figure 6:**
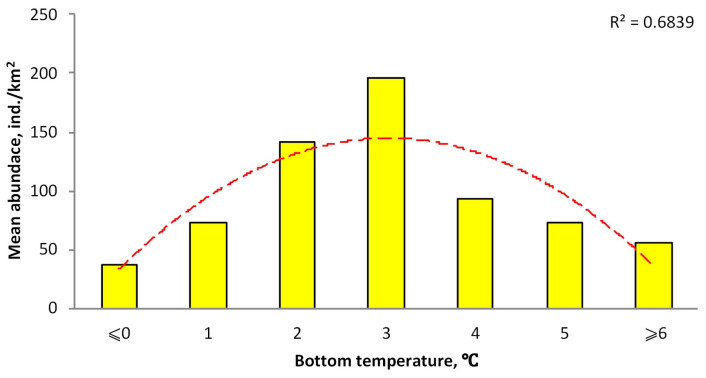
Catch distribution of the Aleutian skate *Bathyraja aleutica* according to bottom temperature in Alaskan waters: (yellow bar) mean value, (dash line) smoothed trend (R^2^ = 0.6839).

Our data on bottom temperatures characterizing the thermal regime of the Aleutian skate habitat are limited to Alaskan waters (eastern part of the Bering Sea, Gulf of Alaska, and Aleutian Islands). They indicate that this species occurs at bottom temperatures of −1.8 to 10 °C (Figure 6). Maximum catches were observed in the range of 2–4 °C. At the same time, with an increase in temperature from negative values to 3 °C, catches grew, and then with its increase, they began to decrease. Thus, bottom temperatures of 2–4 °C should be considered as an optimal temperature for the Aleutian skate in the northeastern Pacific.

### 3.3. Depth Distribution

**Figure 7:**
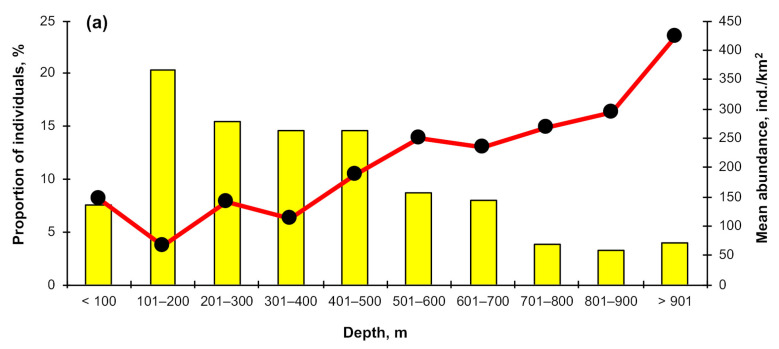

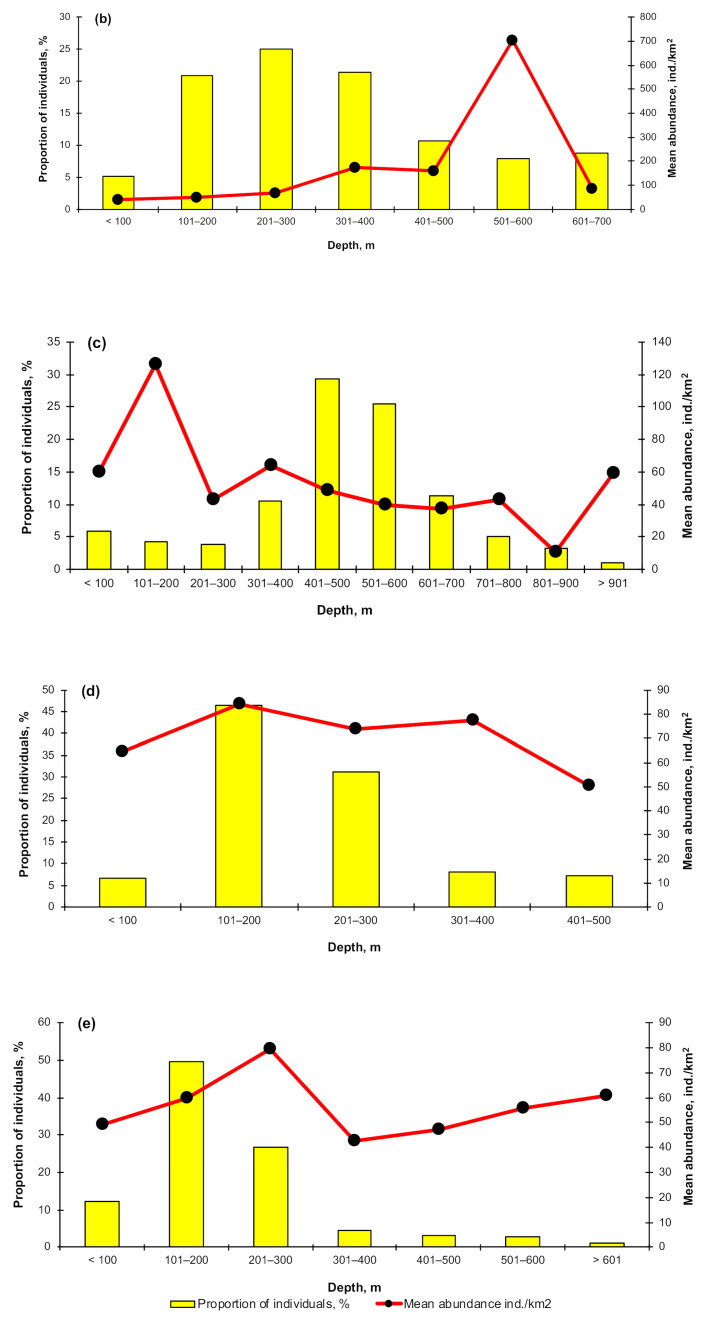
Depth distribution of the Aleutian skate *Bathyraja aleutica* from different regions of its range: (**a**) Bering Sea, (**b**) NWPO, (**c**) Sea of Okhotsk; (**d**) Aleutian islands, (**e**) Gulf of Alaska.

The depth distribution of the Aleutian skate in different parts of its range (NWPO, the Sea of Okhotsk, the Bering Seas, the Aleutian Islands region, the Gulf of Alaska) varied significantly (pairwise two-sided Wilcoxon test with Holm *p*-value adjustment, α = 0.05). In the Bering Sea (Figure 7a), the average catch and occurrence of this species at depths of 100–500 m varied between 66–188 ind./km^2^ and 15–20%, respectively. This area was characterized by an increase in average catch with depth. In the NWPO (Figure 7b), the maximum occurrence of the Aleutian skate was observed at depths of 100–400 m (21–25%), and the highest rates of average catch were recorded within the depth range of 500–600 m (706 ind./km^2^). In the Sea of Okhotsk (Figure 7c), the maximum occurrence was observed at depths from 400 to 600 m (26–30%), while the maximum value of the average catch (126 ind./km^2^) was typical for depths of 100–200 m. For the waters off the Aleutian Islands (Figure 7d), the maximum density of accumulations and catch values were recorded at depths of 100–300 m (78% and 74–84 ind./km^2^, respectively). Maximum occurrence and catches of the Aleutian skate in the Gulf of Alaska (Figure 7e) were recorded in the depth range of 100–300 m (27–49% and 60–80 ind./km^2^, respectively).

**Figure 8:**
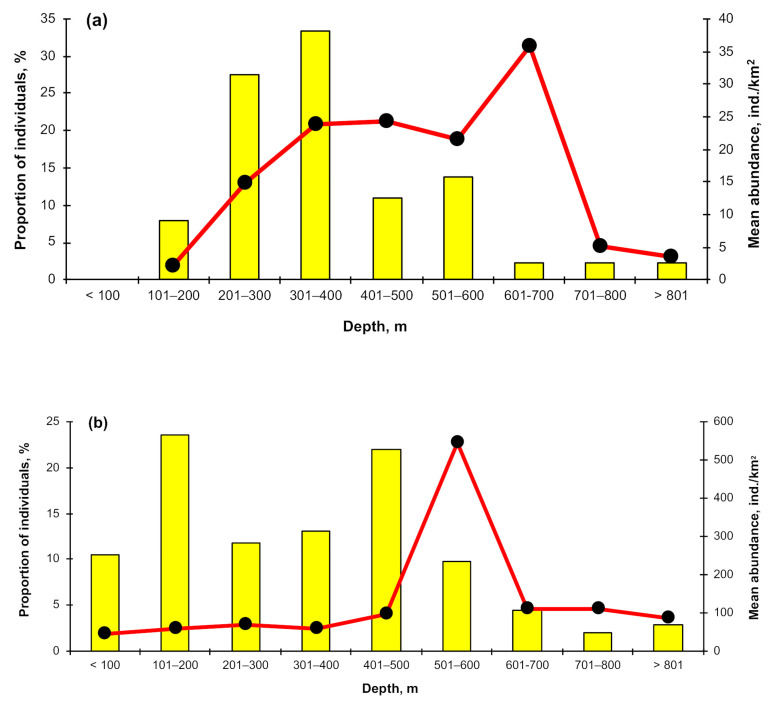

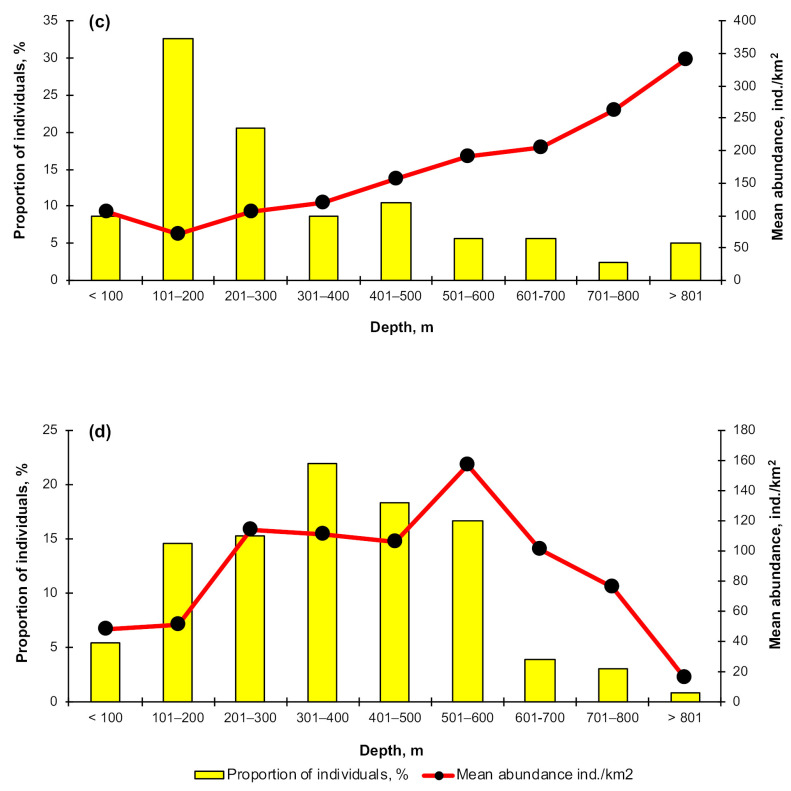
Depth distribution of the Aleutian skate *Bathyraja aleutica* across the North Pacific by season: (**a**) December–February, (**b**) March–May, (**c**) June–August, (**d**) September–November.

The depth distribution of Aleutian skates across the entire area of research in summer statistically differed from that in other seasons; additionally, the depth distribution in winter was significantly different from that observed in the fall (pairwise two-sided Wilcoxon test with Holm *p*-value adjustment, α = 0.05). Depth distributions during fall and spring, and during spring and winter were not significantly different based on the same test. In winter, the bulk of skates was observed in the range of 200–400 m (Figure 8a), where average catches varied between 15–24 ind./km^2^, and the occurrence was between 28–33%. The maximum catch value (36 ind./km^2^) was recorded at a depth of 600–700 m. In spring (Figure 8b), occurrence slightly increased for depths of 100–200 m and 400–500 m (23–24%), and the concentrations with maximum density (550 ind./km^2^) were recorded at depths of 500–600 m. Summer (Figure 8c) was characterized by a higher occurrence of the Aleutian skate at lower depths of 100–300 m (21–33%) and a maximum density at depths over 800 m, where the average catches amounted to 341 ind./km^2^. This period was characterized by an increase in the value of catches with depth. The autumn period (Figure 8d) was characterized by a maximum occurrence of the Aleutian skate at depths of 300–600 m (17–22%) and maximum catches at depths of 500–600 m (157 ind./km^2^).

**Figure 9:**
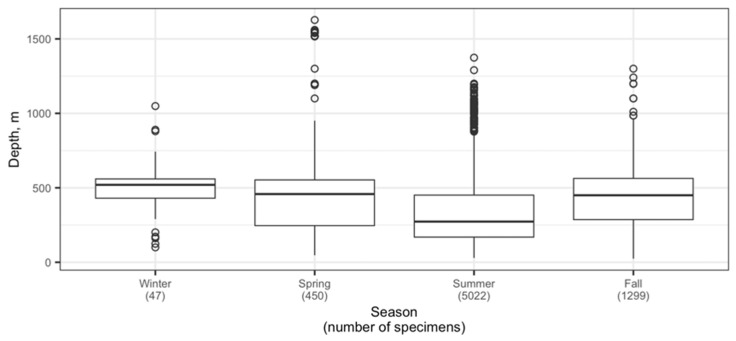
Boxplots of seasonal changes in the depth distribution of the Aleutian skate *Bathyraja aleutica* in the North Pacific Ocean. Circles denote outliers.

The data presented in Figure 9 provide a more detailed picture of the changes in the depth distribution of the Aleutian skate by year, characterized by a narrow bathymetric range of occurrence in the winter (December–February) and early spring (March–April). In general, across its entire range, the maximum average depths (523–550 m) were typical for January and December, and the minimum average depths fell in February and November (264–265 m). The minimum capture depths (24–50 m) were recorded from May to October, while the maximum depth of capture (1627 m) was recorded in May.

### 3.4. Length and Weight

**Figure 10:**
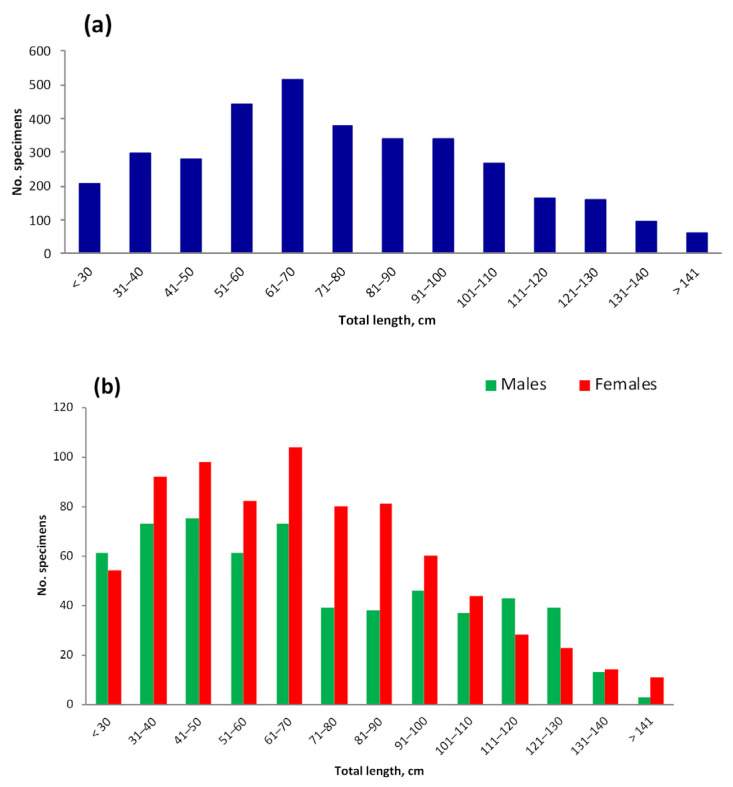
Total length of the Aleutian skate *Bathyraja aleutica* in catches in the North Pacific Ocean: (**a**) both sexes (M = 66.52 cm, 906 ind.), (**b**) males (M = 67.94 cm, 364 ind.) and females (M = 63.69 cm, 341 ind.).

The size distribution of Aleutian skate catches was represented by individuals’ TL range of 10–170 (mean 74.97) cm, with a predominance (64.2%) of individuals having TL 50–100 cm (Figure 10a). Although visual analysis of the length histograms suggests a difference in length between males and females (Figure 10b), this difference was not statistically significant (two-sided Welch’s *t*-test, α = 0.05). Catches were characterized by a numerical predominance of individuals of the size group up to 70 cm (57% for males and 59% for females). The weight of females varied in the range of 0.04−21.55 (mean 3.65) kg, and males in catches had a body weight of 0.04−18.2 (mean 4.1) kg (significant difference, Welch’s *t*-test, α = 0.05).

The size of the Aleutian skate differed markedly between regions (Table 2). Note that the data on skate length is very limited in our database for the regions of Aleutian Islands, eastern Bering Sea, and Gulf of Alaska. Of all regions across its range, the Sea of Okhotsk and British Columbia waters showed the largest maximum mean length (significant differences with both NWPO and western Bering Sea in both cases, two-sided Welch’s *t*-test, α = 0.05). The smallest mean lengths were recorded from the NWPO and the western Bering Sea.

**Figure 11:**
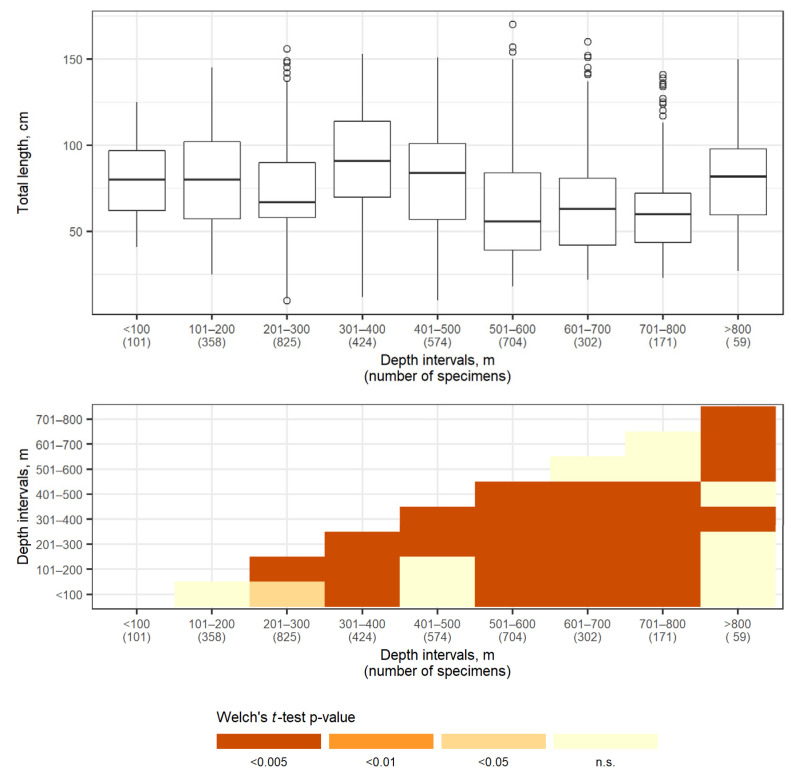
Distribution of the total length of Aleutian skate *Bathyraja aleutica* (cm) across selected depth intervals (m): box-plots of total length distribution (**upper** panel), circles denote outliers, and results of length distribution comparison at different depths by two-sided Welch’s *t*-test with Holm *p*-value adjustment (**lower** panel).

The size composition of the Aleutian skate for the entire study area differed at various depths (Figure 11). Minimum mean values of length were recorded at depths of 100–300 m and 500–800 m, with maximum values being within depth ranges of 200–300 m and over 800 m. The widest size range was characteristic for depths of 200–600 m. Figure 11b shows results of pairwise Welch’s *t*-tests between length samples from different depth intervals. Significant differences in length distribution (*p* < 0.05) were observed between most of the selected depth ranges.

**Figure 12:**
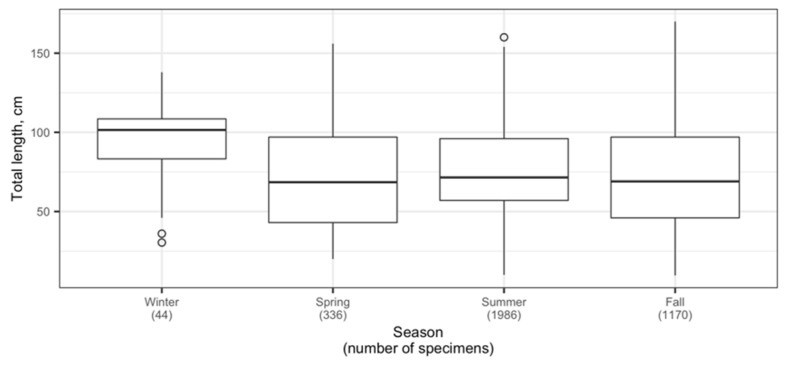
Changes in total length of the Aleutian skate *Bathyraja aleutica* from the North Pacific by time of year. Circles denote outliers.

The size composition of the Aleutian skate in the North Pacific was characterized by changes during the year (Figure 12). Total lengths of specimens caught in winter (December–February) were generally larger compared to other seasons, but note the relatively small sample size (44 specimens) which makes the result less robust. The proportion of smaller skates was larger in spring (March–May), summer (June–August), and fall (September–November) compared to winter. Overall, the total length of Aleutian skates caught in different seasons differed significantly, with the exception of spring and fall, for which the difference in total length was not significant (pairwise two-sided Welch’s *t*-test, α = 0.05).

According to the values of the linear coefficient *a* and the exponent *b*, the equations of the length–weight relationship (LWR) of Aleutian skates differed considerably between regions (Table 2), but not necessarily significantly (not shown in table). For males, no significant difference in both coefficients was observed between regions. However, for females, both coefficients showed significant differences across all considered regions (*p* < 0.05). When both sexes were considered together, no significant difference in either coefficient was observed between regions.

Analysis of the magnitude of the exponent of the LWR equation showed that males from the western Bering Sea and the Sea of Okhotsk are characterized by a growth pattern close to isometric (3.04–3.06). A similar growth pattern was also inherent in Aleutian skates in the Sea of Okhotsk when sex was not considered (3.03). For the female Aleutian skate and the bulk of skates from the waters off British Columbia, negative allometric growth was observed (2.82–2.95). From the remaining analyzed regions, individuals of the Aleutian skate showed positive allometric growth.

### 3.5. Sex Ratio and Condition Factor

**Figure 13:**
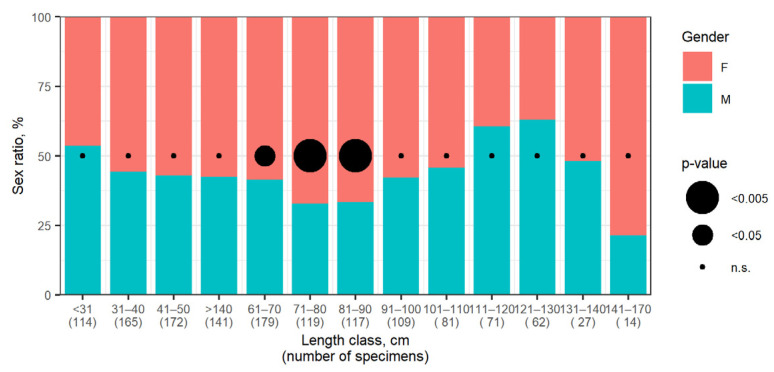
Sex ratios by size class of the Aleutian skate *Bathyraja aleutica* from the North Pacific. Black circles show the test of proportion (1:1) *p*-value.

The male-to-female ratio was close to 1:1 for small skates (TL < 30 cm) (53% of males), whereas females dominated in the 30–110 cm size group (54–68%), and then males predominated in the 110–130 cm size group (60–63%). An almost equal sex ratio was observed in the 130–140 cm size group, and among the largest individuals (TL > 140 cm), females dominated (79%). The male-to-female ratio did not significantly differ from 1:1 in all length classes with the exception of 61–70, 71–80, and 81–90 cm, where females significantly predominated (test of proportion 1:1, α = 0.05, Figure 13).

**Figure 14:**
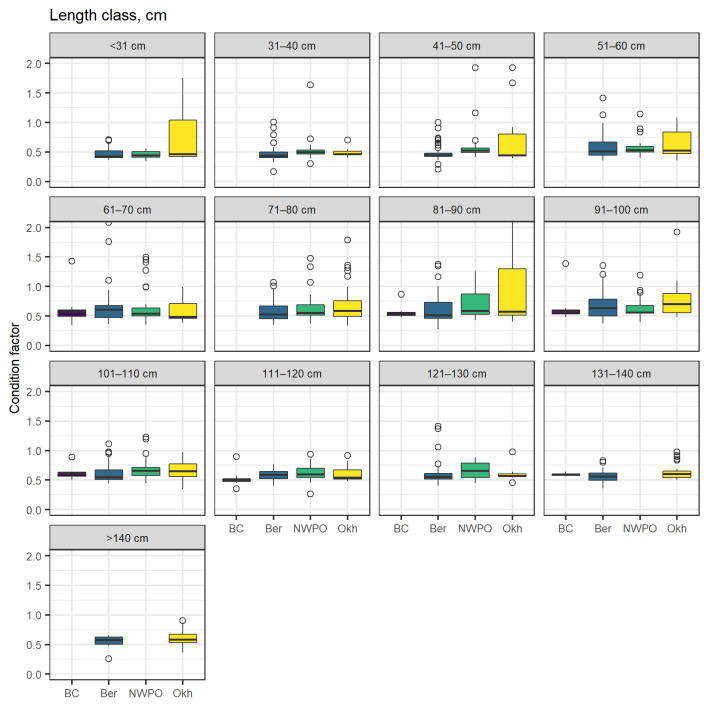
Boxplots of condition factor of the Aleutian skate *Bathyraja aleutica* of different size classes from different locations; BC—the waters off British Columbia, Ber—the Bering Sea, NWPO—the northwestern Pacific Ocean, Okh—the Sea of Okhotsk. Circles denote outliers.

Condition factor values, which characterize the ratio between length and body weight, in the Aleutian skate varied between 0.16 and 4.4 (mean 0.61) in the North Pacific as a whole. For the skates from the Bering Sea, it was in the range of 0.16 to 2.23 (0.58), the Sea of Okhotsk—0.34−1.42 (0.59), NWPO—0.26−4.40 (0.63), and in the Pacific waters of Canada—0.34–1.42 (0.59). In all areas considered, the relationship between total length and condition factor was not notably observed, although in the Bering Sea, the Sea of Okhotsk, and the NWPO the variability in condition factor for middle size classes (total length between 60 and 100 cm) was considerably higher than for smaller and larger individuals. Comparison of the condition factor within selected length classes across four regions for which sufficient data were available revealed an apparent increase of condition factor from east to west (the condition factor was lowest in the waters off British Columbia and the Bering Sea and highest in the northwest Pacific Ocean and the Sea of Okhotsk) for virtually all length classes. However, only in a few cases was this difference significant (pairwise two-sided Wilcoxon test with Holm *p*-value adjustment, α = 0.05) (Figure 14).

**Figure 15:**
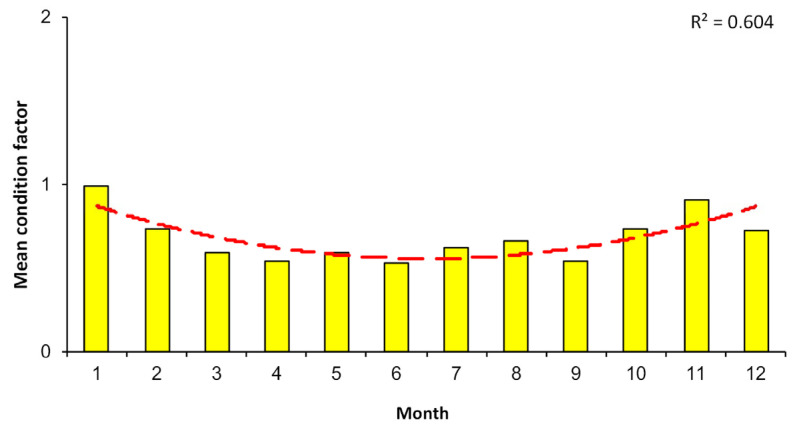
Changes in condition factor of the Aleutian skate *Bathyraja aleutica* from the North Pacific across the year; (yellow bar)—mean value, (dash line) smoothed trend (R^2^ = 0.604).

The condition factor of the Aleutian skate is subject to some seasonal dynamics (Figure 15). Its maximum value typically occurred in January (0.99), from January to June it decreased, and from July to November it increased with a slight decrease in December.

### 3.6. Catch Dynamics

**Figure 16:**
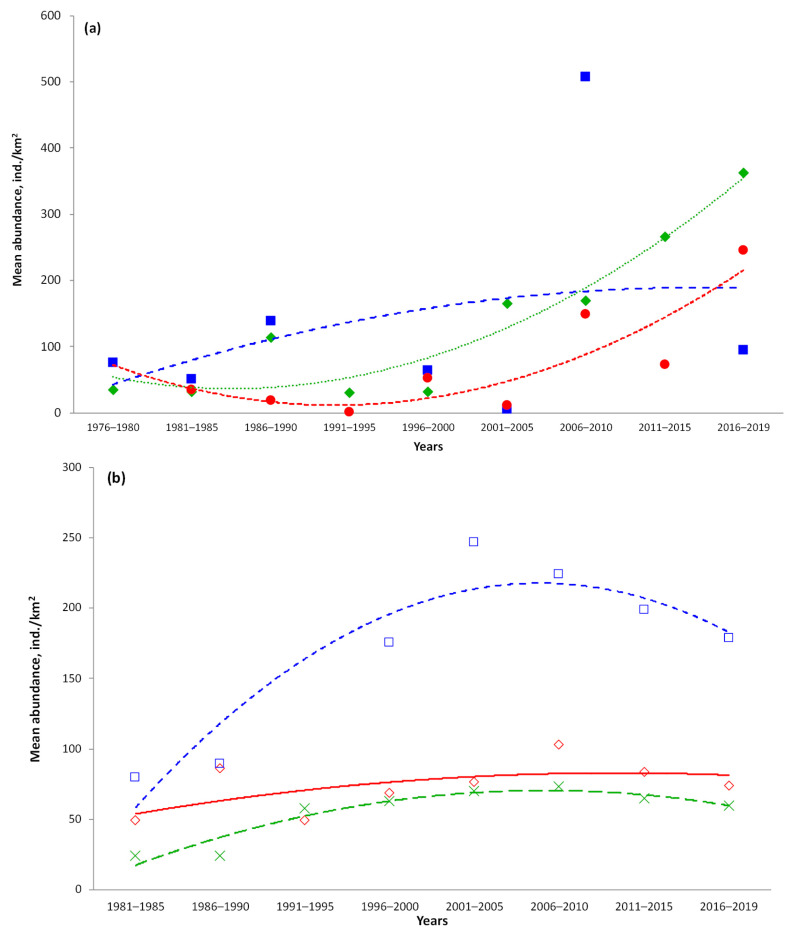
Region-specific dynamics of the abundance of the Aleutian skate *Bathyraja aleutica* from the North Pacific by year: (**a**) Russian waters and (**b**) Alaskan waters. Mean values and trends: (♦, ···) Western Bering Sea; (■, - -) NWPO; (●, ---) Sea of Okhotsk; (◇) Aleutian Islands; (x, ---- ) Gulf of Alaska; (□, ···) Eastern Bering Sea.

According to the bottom-trawl-survey catch data in the western Bering Sea, the catch rates of Aleutian skate during the entire period of research showed an upward trend, indicating a progressive increase in its abundance (Figure 16a). From the Sea of Okhotsk, there was a decrease in catches until the mid-1990s, after which their growth rates demonstrated positive trends. In the NWPO, until the early 2000s, catches showed a weak positive trend, after which their values varied little.

In the eastern Bering Sea, Aleutian skate catches steadily increased until the early 2000s, after which they also gradually began to decline (Figure 16b). Catches off the Aleutian Islands during the entire period of research showed a weak but positive trend. In the Gulf of Alaska, the catches gradually increased until the second half of the 2000s, after which they also gradually declined.

## 4. Discussion

### 4.1. Spatial Distribution

The spatial distribution of *B. aleutica* in particular areas of the North Pacific have been studied relatively well [12,39,45,51,64,65]. Our data generally do not contradict the previously obtained information on the spatial catch distribution of Aleutian skates in the north Pacific waters off the northern Kuril Islands, southeastern Kamchatka, Alaska, and British Columbia. In addition, the data presented fill a substantial the gap in our knowledge regarding the distribution of the Aleutian skate in other areas of the North Pacific and provide a more complete picture of its distribution throughout the species’ range.

Thus far, seasonal patterns in the spatial distribution of the Aleutian skate have not been described. The catch distribution of this species in the north Pacific waters off the northern Kurils and southeastern Kamchatka during the summer and autumn of two adjacent years (1996–1997) were presented [39]. Our data allowed us to trace seasonal changes in the pattern of Aleutian skate distribution throughout its entire range.

Long-term changes in the spatial distribution of the Aleutian skate have not yet been analyzed. Our data allow us not only to assess the nature of changes that have occurred in the spatial distribution of this species over the past 40 years, but also to trace fluctuations in its abundance across various areas of the North Pacific during this period.

### 4.2. Depth Distribution

It is known that the Aleutian skate in the North Pacific occurs at depths from 15 to 1602 m [21,28,38,52,66,67]. At the same time, the optimal depth ranges for this species are 400–800 and 1400–1600 m [31]. According to our data, the species has been recorded in catches at depths of 14–1627 m, which somewhat expands the known bathymetric range of its habitat.

The Aleutian skate belongs to the lower bathyal (mesobenthic) species and it is constantly found throughout the year at depths of 500 (1000)–2000 (3000) m [36]. A characteristic feature of the lower bathyal skates is a very wide bathymetric habitat range caused by relatively homogeneous living conditions at great depths. As a result, in summer, the majority of individuals of the Aleutian skate adhere to depths of 250–500 m, and in winter they live at depths of 470–700 m [36]. The data obtained by us indicate that during the year the main concentrations of the Aleutian skate were within the depth range of 100–600 m. For all periods, except winter, the occurrence of individual Aleutian skates at depths shallower than 100 m was observed, which may be due to their dispersal during the feeding period in a wide bathymetric range in order to maximize their feeding ground and thereby reduce the level of intraspecific competition. Spring periods were characterized by an increase in the number of Aleutian skates at depths shallower than 100 m, which is probably explained by the beginning of the summer feeding migration. In contrast to [33], our materials indicate that during the summer period, the majority of skates feed at depths of 100–300 m, and not 250–550 m. The autumn was characterized by a shift of most individuals to depths of 300–600 m, which may mean the beginning of over-wintering.

According to literature data, in the western Bering Sea, the Aleutian skate occurs within the range of 157–1100 m [33]. The data obtained by us are very consistent with [33], since the main concentrations of this species were formed at depths of 100–500 m. However, our data indicate shallower depths of occurrence of this species in the Bering Sea in comparison with previously published data [33] (minimum depths of 24 m versus 157 m, respectively).

In the Pacific waters off the northern Kuril Islands and southeastern Kamchatka, in the period from May to October, the Aleutian skate occurs at depths from 75 to 800 m, while its main aggregations are observed at depths of 175–315 m [39]. According to [4], about 60% of the Aleutian skate in this area from spring to autumn are concentrated at depths of 250–600 m. In the Pacific waters off Japan, the range of occurrence of this species is within the range of 600–1210 m [33]. Our data show that the Aleutian skate is the most frequently considered species in the NWPO occurring in the depth range from 100 to 400 m, and their schoolings with maximum density are formed at depths of 500–600 m, which somewhat contradicts previously published data. The reason for this may be associated with the fact that the Pacific waters off Japan, the Kuril Islands, and eastern Kamchatka are characterized by a range of different biotopes, including both areas with a steep continental slopes and well-defined shelves, within which the nature of the depth distribution of *B. aleutica* can vary greatly. Our data represent the combined information from all waters of the northwestern Pacific, and therefore eliminate the differences inherent in particular areas.

### 4.3. Distribution Depending on Bottom Temperatures

Published data on bottom temperatures of the Aleutian skate habitat in the North Pacific indicate its eurythermicity. The bottom temperature range at which it occurs is quite wide: from −0.3 to 3.5 °C [34]. For example, in the Pacific waters off Kamchatka and the Kuril Islands, it was observed at bottom temperatures from 0 to 4 °C with maximum schoolings within the range of 3–4 °C [45], while off the west coast of the USA, captures were recorded within the range of bottom temperatures between 5.2 to 5.9 °C [22]. Our data indicate that in the waters off Alaska, the Aleutian skate occurs at bottom temperatures from −1.2 to 9.2 °C, and forms maximum concentrations at bottom temperatures around 3 °C. The data obtained on the thermal regime indicate a greater eurythermicity of the Aleutian skate than previously thought.

### 4.4. Length and Weight

Bottom trawl catches in the Pacific waters off the northern Kuril Islands and southeastern Kamchatka included Aleutian skates with TL 22–134 (mean 82.4) cm, with a predominance of individuals with a TL between 106 and 110 cm [45]. In the western Bering Sea this species was 22–134 cm long [56], while in the waters off Alaska its maximum length was 154 cm [43]. Our data showed that, in general, in the North Pacific, the Aleutian skate was represented in catches by individuals with TLs within the range of 9.6−170 cm, with the predominance of skates being 50–100 cm long.

The relationship between the length and body weight of the Aleutian skate is described for several regions (Table 2). A comparison of the exponents of this relationship obtained by us and previously published [45,56] values shows that they differed significantly, both in individuals of different sexes and from different areas. For males in the western Bering Sea, a positive allometric growth pattern was previously shown [56]. According to our data, from this area, as well as in the Sea of Okhotsk, males of the Aleutian skate show a growth pattern close to isometric. In the NWPO and British Columbia waters, they were also characterized by positive allometric growth. Positive allometric growth was previously shown for females of the Aleutian skate in the western Bering Sea [56]. We observed a similar growth pattern not only in this area, but also from the rest of the study areas, with the exception of the waters off British Columbia, where it was negative allometric. In general, positive allometric growth was previously observed for both sexes in the western Bering Sea and the Pacific waters off the northern Kurils and southeastern Kamchatka [45,56], which is confirmed by our data. In the Sea of Okhotsk their growth is close to isometric, and in the waters off British Columbia it has a negative allometric characteristic. The reasons for this difference may be associated with the fact that previously published data characterizing the LWR of the Aleutian skate was obtained during a short period of time (summer–autumn period), when its individuals were in a feeding condition. Our data were obtained in a more temporally protracted period (most of the year), when individuals of this species may be in varying physiological states. The obvious difference in the considered feature of the skates from British Columbia waters has not yet found a reasonable explanation. A smaller value of the LWR equation exponent indicates a lower condition factor [68,69], which may be due to suboptimal living conditions on the outskirts of its range (temperature, oxygen content, food availability, etc.).

### 4.5. Sex Ratio and Condition Factor

Data on the sex ratios of Aleutian skates across various size classes are few. Some authors [52,53] suggest that the sex of the Aleutian skate becomes distinguishable (claspers begin to be visible) only when TL > 70 mm is reached, below which all embryos resemble females, while in embryos TL > 70 mm, the sex ratio is approximately 1:1. An equal sex ratio of embryos is noted in egg capsules [36], while equal sex ratios are also characteristic of natural populations. However, females predominate in the older age groups of all Far Eastern skates due to earlier maturation of males, their shorter lifespan and lower growth rates [37]. Larger sizes of females are of adaptive importance for ensuring greater fecundity of the population [70]. It was previously shown [45] that in the Pacific waters off the northern Kuril Islands and southeastern Kamchatka, females predominate among the smallest individuals of the Aleutian skate (TL < 50 cm). They also dominate in the size classes 60–90 cm and >120 cm, while an equal sex ratio was observed in the size class of 110–120 cm. According to our data, males are more numerous among skates with TL < 30 cm, females dominate in the 30–110 cm size class, the sex ratio is almost equal in the 130–140 cm size class, and females predominate among individuals TL > 140 cm. Thus, our generalized data for the entire North Pacific on the sex ratio in different size classes of the Aleutian skate fully correspond to the previously published information for the Russian Far Eastern seas [37], and differ slightly from those for the Pacific waters off the northern Kurils and southeastern Kamchatka [45]. The reason for these differences probably include a number of limitations in the collection of data compared (bathymetric range, summer-autumn season, local geographical area, etc.).

Information regarding the condition factor of Aleutian skates is available only for the western Bering Sea [40]. Thus, according to these data, for male Aleutian skates, the maximum condition factor from different seasons was 8.8, and that for females was 9.6. According to our data, the maximum condition factor for the western Bering Sea was 4.4, while 95% of the individuals from all study areas had a condition factor < 1%. For the Aleutian skate, Alaska skate *B. parmifera*, Okhotsk skate *B. violacea*, and Pacific sleeper shark *Somniosus pacificus*, a general trend of increased condition factor in the autumn–winter period and a decrease in its value by the summer has been revealed [71,72,73], as well as in this study, which may be due to similar ecological and biological conditions of these species (ecological niche, biotopes, migrations, etc.). This trend indicates a feeding migration in the spring–summer from greater to shallower depths, and in the autumn–winter in the opposite direction.

### 4.6. Catch Dynamics

Among all the deep-water skates of the Russian Far Eastern seas, the Aleutian skate is the third in terms of biomass (87.7 thousand tons), after the Alaska skate *B. parmifera* and the Matsubara skate *B. matsubarae*, with biomasses of 273.6 and 117.6 thousand tons, respectively [12]. In the Russian part of our study area, the data on the catch dynamics of the Aleutian skate were presented only for the Pacific waters off the northern Kurils and southeastern Kamchatka for the period 1993–2000 [45], during which there was a fairly pronounced positive trend. The data presented by us for the NWPO differ from the results of the above-mentioned publications, demonstrating a possible downward trend in catch rates in the early 1990s and a rise only by the early 2000s. This difference may be due to the fact that the Pacific waters off southeastern Kamchatka and the northern Kurils are only part of our NWPO area, which, in addition, also includes the Pacific waters off Japan and northeastern Kamchatka.

Data on the Aleutian skate dynamics of the biomass in the Gulf of Alaska in the period from 2000 to 2019 [74] was quite comparable with our data on the dynamics of catch rates of this skate (an increase by 2005–2010, followed by a fall). When comparing the data obtained for the eastern Bering Sea and the Aleutians [75], differences were revealed, e.g., the USA data indicated an increase in biomass between 2005 and 2010, while our data showed a drop in catches, then since 2011 there was a positive trend in both the USA data and ours. The reasons for the differences between our and previously published data are still unclear and may be related to different methodological approaches to data analysis.

Currently, targeted fishing for skates in the Russian Far Eastern waters has not been developed, with catches occurring as by-catch of other target fisheries [10,11]. Historically, catch surveys did not separate skates by species, and they were not retained and utilized, but discarded after capture [76,77,78]. This situation has changed in recent years when several Russian companies started to export skate wings to China and the British Virgin Islands [10,11]. They also may be profitably marketed in Japan and Korea. In waters of the Russian Far East, 11.4–11.7 Kt were recommended for harvesting in the 1990s, 7.2–11.9 Kt in the 2000s, 11.2–14.0 Kt in the 2010s, and 11.2–11.3 Kt in the 2020s [13]. Since the fishing of deep-water skates, including the Aleutian skate, is currently developing in the North Pacific, an important role in their management should consider regular stock assessments and monitoring of the populations. The results of the present study have shown that in the western Bering Sea and the Sea of Okhotsk, the abundance of the Aleutian skate is increasing, to the extent that it would not require a reduction in the efforts of deep-water groundfish fisheries. From other areas of Aleutian skate habitat (the Pacific waters off the Kuril Islands and eastern Kamchatka, the eastern Bering Sea, the Aleutian Islands, and the Gulf of Alaska), in recent years there has been no increase in their abundance. Thus, the current state of its populations in these areas may require a reduction in fishing effort in deep-water groundfish fisheries, which will help to avoid overfishing and to contribute to the conservation of the species.

## 5. Conclusions

The results of the analysis of long-term data on the Aleutian skate *Bathyraja aleutica* records in the North Pacific Ocean allowed for obtaining the comprehensive information about species’ spatial and vertical distributions, dynamics of abundance, and size composition in this area. This species is most abundant off the eastern Bering Sea slope, the central Aleutian Islands, in the Pacific waters off southeastern Kamchatka and the northern Kuril Island, and northeastern Sakhalin. The main habitat depths of the Aleutian skate are 100–600 m with shift to greater depths in cold period of the year and to shallower depths in warm seasons. *B. aleutica* prefers bottom temperatures around 3 °C. Bottom trawl catches are presented by individuals with total length ranged 9.6 to 170 cm with a predominance of skates 50–100 cm long. Sizes of male and female Aleutian skates were almost not different. Based on the analysis of *B. aleutica* catch rate dynamics it can be noted that the current state of its populations in the Pacific waters off the Kuril Islands and eastern Kamchatka, the eastern Bering Sea, the Aleutian Islands, and the Gulf of Alaska, requires a reduction in fishing effort in deep-water groundfish fisheries. At the same time, in the western Bering Sea and the Sea of Okhotsk, the abundance of this species is increasing, and therefore a reduction in the efforts of deep-water groundfish fisheries would not require. Such fishery management measures will help to avoid overfishing and to conserve the species considered.

## Figures and Tables

**Table 1:** Description of the data used for the analysis of spatial and vertical distribution of the Aleutian skate *Bathyraja aleutica* in the North Pacific, 1893–2021.

Data Type	No. Records	Data Source/ Organization	Link to Data Source
Scientific bottom-trawl surveys	3356	Pacific branch of Russian Federal Research Institute of Fisheries and Oceanography (TINRO), Vladivostok, Russia	The data received upon request on 1 October 2022
Scientific bottom-trawl surveys	3010	Alaska Fisheries Science Center (AFSC), National Oceanic and Atmospheric Administration (NOAA), Seattle, WA, USA	https://www.fisheries.noaa.gov/alaska/commercial-fishing/alaska-groundfish-bottom-trawl-survey-data accessed on 3 October 2022
Scientific bottom-trawl surveys	47	Northwestern Fisheries Science Center National Oceanic and Atmospheric Administration—NWFSC NOAA, Seattle, WA, USA.	https://www.webapps.nwfsc.noaa.gov/data/map accessed on 3 October 2022
Observations on commercial fishing vessels	8567	Alaska Fisheries Science Center (AFSC), National Oceanic and Atmospheric Administration (NOAA), Seattle, WA, USA	https://www.fisheries.noaa.gov/resource/map/spatial-data-collected-groundfish-observers-alaska accessed on 3 October 2022
Museum collection	40	Fish Collection of Hokkaido University—National Museum of Nature and Science, Hokkaido University, Sapporo, Hokkaido, Japan	https://www.gbif.org/ru/occurrence/search?dataset_key=848ae956-f762-11e1-a439-00145eb45e9a&taxon_key=2420280 accessed via GBIF.org on 3 October 2022
Institution collection	18	Ichthyology collection of the California Academy of Sciences (CAS), San Francisco, CA, USA	https://www.gbif.org/ru/occurrence/search?publishing_org=66522820-055c-11d8-b84e-b8a03c50a862&taxon_key=2420280 accessed via GBIF.org on 3 October 2022
Scientific bottom-trawl surveys	1	Pacific Biological Station (PBS), Fisheries and Oceans Canada, Nanaimo, BC, Canada	https://www.gbif.org/ru/occurrence/search?dataset_key=310dbb60-e369-4ec2-a6d8-8e4de843b208&publishing_org=c98a8d0d-cab2-4506-8c0f-c91a5e4996b9&taxon_key=2420280 accessed via GBIF.org on 3 October 2022
Scientific bottom-trawl surveys	128	Pacific Biological Station (PBS), Fisheries and Oceans Canada, Nanaimo, BC, Canada	https://www.gbif.org/ru/occurrence/search?dataset_key=9fcd8de5-a2b6-4c72-b863-debe63ee3a72&publishing_org=c98a8d0d-cab2-4506-8c0f-c91a5e4996b9&taxon_key=2420280 accessed via GBIF.org on 3 October 2022
Scientific bottom-trawl surveys	44	Pacific Biological Station (PBS), Fisheries and Oceans Canada, Nanaimo, BC, Canada	https://www.gbif.org/ru/occurrence/search?dataset_key=48060741-47d3-4093-86e6-2ec484323cc5&publishing_org=c98a8d0d-cab2-4506-8c0f-c91a5e4996b9&taxon_key=2420280 accessed via GBIF.org on 3 October 2022
Scientific bottom-trawl surveys	46	Pacific Biological Station (PBS), Fisheries and Oceans Canada, Nanaimo, BC, Canada	https://www.gbif.org/ru/occurrence/search?dataset_key=3d4cac0a-7441-4099-8eed-8a270d522f8f&publishing_org=c98a8d0d-cab2-4506-8c0f-c91a5e4996b9&taxon_key=2420280 accessed via GBIF.org on 3 October 2022
Scientific bottom-trawl surveys	4	Pacific Biological Station (PBS), Fisheries and Oceans Canada, Nanaimo, BC, Canada	https://www.gbif.org/ru/occurrence/search?dataset_key=f2189339-9098-4e0e-98d4-9c72e5e5f96d&publishing_org=c98a8d0d-cab2-4506-8c0f-c91a5e4996b9&taxon_key=2420280 accessed via GBIF.org on 3 October 2022
Scientific bottom-longline surveys	17	Pacific Biological Station (PBS), Fisheries and Oceans Canada, Nanaimo, BC, Canada	https://www.gbif.org/ru/occurrence/search?dataset_key=1677f287-3666-49b8-90f2-12a63ca58438&publishing_org=c98a8d0d-cab2-4506-8c0f-c91a5e4996b9&taxon_key=2420280 accessed via GBIF.org on 3 October 2022
Scientific bottom-longline surveys	7	Pacific Biological Station (PBS), Fisheries and Oceans Canada, Nanaimo, BC, Canada	https://www.gbif.org/ru/occurrence/search?dataset_key=e908605e-fb40-4db1-bff7-de1c241284a9&publishing_org=c98a8d0d-cab2-4506-8c0f-c91a5e4996b9&taxon_key=2420280 accessed via GBIF.org on 3 October 2022
Museum collection	2	Vertebrate, zoology, fishes collections of Smithsonian National Museum of Natural History—NMNH, Washington, DC, USA.	https://collections.nmnh.si.edu/search/fishes/ accessed on 3 October 2022
Museum collection	6	Fish collection of the Canadian Museum of Nature, Ottawa, ON, Canada	http://ipt.nature.ca/resource?r=cmn_fish&v=1.124 accessed on 3 October 2022
Museum collection	66	Ichthyology collection of Burke Museum of Natural History and Culture (BMNHC), Seattle, WA, USA	https://www.burkemuseum.org/collections-and-research/biology/ichthyology/collections-database/results.php?l=100&o=0&f=&g=&s=17h~IymyNd1yIp&d=gpA&w=PhIyNl1XhPYMY0j1yI1Kp0YndhYPhSh|b3NhsPYMY0jld1B0&wo=PhIyNl1XhPYjXhlE1Yj1yI1KpYndhYPhSh|b3NhsPYjXhlE1Yjld1B accessed on 3 October 2022
Museum collection	2	Ichthyology collection of Muséum national d’Histoire naturelle (MNHN), Paris, France	https://obis.org/taxon/271506 accessed via OBIS.org on 3 October 2022
Institution collection	1	Ichthyology collection University of Kansas Biodiversity Institute & Natural History Museum (KUBI), Lawrence, KS, USA	https://obis.org/taxon/271506 accessed via OBIS.org on 3 October 2022
Museum collection	1	Fish collection of the National Museum of Nature and Science (NMNS), Tokyo, Japan	https://obis.org/taxon/271506 accessed via OBIS.org on 3 October 2022
Institution collection	12	Texas Cooperative Wildlife Collection (TCWC), Department of Wildlife and Fisheries Sciences, Texas A&M University, College Station, TX, USA	https://www.gbif.org/ru/occurrence/search?publishing_org=2b8e48b0-812d-11de-86fe-b8a03c50a862&taxon_key=2420280 accessed via GBIF.org on 3 October 2022
Institution collection	1	Marine Vertebrate Collection of the Scripps Institution of Oceanography (SIO), San Diego, CA, USA	https://sioapps.ucsd.edu/collections/mv/collection/94-205/?q=Bathyraja+aleutica accessed on 3 October 2022
Museum collection	1	Fish collection of the Museum of the North, University of Alaska (UAM), Fairbanks, AK, USA	http://arctos.database.museum/guid/UAM:Fish:9455?seid=2666501 accessed on 3 October 2022
Total	15,377	22 sources/15 organizations	

**Table 2:** Parameters of length–weight relationships (LWR) of the Aleutian skate *Bathyraja aleutica* by regions within its range (NWPO—the northwestern Pacific Ocean, WBS—the western Bering Sea, Okh—the Sea of Okhotsk, BC—waters off British Columbia, n—number of specimens examined, M—mean value, SE—standard error, TL—total length).

Sex	Area	Parameters of LWR			n	TL, cm		Weight, kg		Source
		a	b	R^2^		Min–Max	M ± SE	Min–Max	M ± SE	
Males	NWPO	3.17 × 10^−6^	3.12	0.99	83	20–145	64.34 ± 2.90	0.04–9.10	2.13 ± 0.27	Our data
	WBS	4.58 × 10^−6^	3.06	0.97	213	11–135	67.30 ± 1.60	0.06–13.98	4.35 ± 0.26	Our data
		2.81 × 10^−6^	3.15	0.99	74	22–130	-	-	-	[56]
	Okh	4.41 × 10^−6^	3.04	0.97	50	20–146	90.51 ± 4.96	0.12–18.20	5.92 ± 0.79	Our data
	BC	3.51 × 10^−6^	3.11	0.94	28	59–137	88.36 ± 4.06	1.02–15.52	4.75 ± 0.70	Our data
Females	NWPO	2.74 × 10^−6^	3.17	0.99	145	24–115	64.83 ± 3.03	0.04–17.60	2.38 ± 0.26	Our data
	WBS	2.83 × 10^−6^	3.16	0.96	207	13–143	66.28 ± 1.25	0.07–18.40	3.45 ± 0.26	Our data
		1.55 × 10^−6^	3.28	0.99	69	28–134	-	-	-	[56]
	Okh	3.23 × 10^−6^	3.12	0.98	61	27–153	85.91 ± 3.78	0.08–21.55	5.48 ± 0.75	Our data
	BC	1.31 × 10^−5^	2.82	0.90	42	52–140	98.26 ± 3.39	0.82–17.15	6.35 ± 0.60	Our data
Both sexes	NWPO	2.61 × 10^−6^	3.20	0.96	356	20–145	74.17 ± 1.54	0.04–18.20	3.89 ± 0.21	Our data
		3.03 × 10^−6^	3.16	-	507	20–128	82.40 ± 0.93	0.06–14.10	4.42 ± 0.30	[45]
	WBS	2.79 × 10^−6^	3.16	0.95	783	10–148	71.92 ± 0.55	0.04–31	4.16 ± 0.15	Our data
		2.13 × 10^−6^	3.21	0.99	143	22–134	-	-	-	[56]
	Okh	5.51 × 10^−6^	3.03	0.93	270	20–170	96.31 ± 1.73	0.08–35	8.21 ± 0.44	Our data
	BC	7.07 × 10^−6^	2.95	0.92	71	10–140	96.83 ± 2.42	0.82–17.15	5.81 ± 0.47	Our data

## Data Availability

Not applicable.
